# LMPred: predicting antimicrobial peptides using pre-trained language models and deep learning

**DOI:** 10.1093/bioadv/vbac021

**Published:** 2022-03-31

**Authors:** William Dee

**Affiliations:** Department of Bioinformatics, School of Biological and Behavioural Sciences, Queen Mary University of London, London E1 4NS, UK

## Abstract

**Motivation:**

Antimicrobial peptides (AMPs) are increasingly being used in the development of new therapeutic drugs in areas such as cancer therapy and hypertension. Additionally, they are seen as an alternative to antibiotics due to the increasing occurrence of bacterial resistance. Wet-laboratory experimental identification, however, is both time-consuming and costly, so *in silico* models are now commonly used in order to screen new AMP candidates.

**Results:**

This paper proposes a novel approach for creating model inputs; using pre-trained language models to produce contextualized embeddings, representing the amino acids within each peptide sequence, before a convolutional neural network is trained as the classifier. The results were validated on two datasets—one previously used in AMP prediction research, and a larger independent dataset created by this paper. Predictive accuracies of 93.33% and 88.26% were achieved, respectively, outperforming previous state-of-the-art classification models.

**Availability and implementation:**

All codes are available and can be accessed here: https://github.com/williamdee1/LMPred_AMP_Prediction.

**Supplementary information:**

Supplementary data are available at *Bioinformatics Advances* online.

## 1 Introduction

Antimicrobial peptides (AMPs) are a set of naturally occurring molecules that exhibit a wide range of functions, including antibacterial, anticancer, antifungal and antihypertensive properties (Bhadra *et al.*, 2018). When used to create therapeutic drugs, peptides are increasingly showing efficacy in terms of treatment in a variety of important areas; ranging from cancer targeting to eliminating bacterial, viral and fungal pathogens (Thundimadathil, 2012).

Current cancer therapies, such as radiotherapy and chemotherapy, often elicit harmful side-effects, and cancer cell resistance to chemotherapeutic agents is a large and growing issue (Rebucci and Michiels, 2013). Peptides have long been used as biomarkers in the detection and diagnosis of specific cancers, such as pancreatic, colorectal and lung (Xiao *et al.*, 2013). However, recently their use has been extended, as they have been shown to bind to specific cancerous sites and so have been used as carriers for targeted drugs (Wang *et al.*, 2014). Certain peptides have also been shown to exhibit an inhibitory effect in cancer cells themselves (Rayaprolu *et al.*, 2013).

Additionally, pathogenic bacteria are more frequently developing multi-drug resistance (Bhadra *et al.*, 2018; World Health Organization, 2014), rendering current antibiotic treatments ineffective. Given that AMPs are endogenous, they have shown a lower likelihood for bacteria to develop resistance to them (Boman, 2003), and so offer a complementary alternative to traditional drugs.

Identification of natural AMPs is therefore becoming increasingly important. However, experimental identification is both time-consuming and costly, hence the need for *insilico* prediction models (Bhadra *et al.*, 2018; Li and Wang, 2016). Previous model-based methods have utilized a range of Machine Learning approaches, including; Hidden Markov Models (Fjell *et al.*, 2007), Fuzzy K-Nearest Neighbour (FKNN; Xiao *et al.*, 2013), Random Forest (RF; Bhadra *et al.*, 2018), Discriminant Analysis (DA; Thomas *et al.*, 2010) and Support Vector Machines (SVM; Manavalan *et al.*, 2017; Tyagi *et al.*, 2013).

Whilst these methods achieved high levels of accuracy, the feature creation steps were often extensive, as they were reliant on generating inputs that represented the biological composition of each peptide sequence. Bhadra *et al.* (2018) performed a survey of existing approaches to summarize the common data pre-processing steps and found that prior research was most likely to use ‘compositional, physicochemical, structural properties, sequence order and the pattern of terminal residues’ in order to create the final feature set. In the case of Manavalan *et al.* (2017), 436 additional features were engineered—20 representing amino acid composition, 400 relating to dipeptide composition, 5 associated with atomic composition and 11 referring to different physicochemical properties.

Compositional metrics, like those described above, fail to take sequence order into account—even though it is integral to the underlying function of peptides. To account for this, some prior approaches used Chou’s pseudo-amino acid composition (PseAAC; Chou, 2001), generating correlation factors between each amino acid, which partly incorporated some relational information regarding sequence order (Meher *et al.*, 2017; Xiao *et al.*, 2013). Similarly, Evolutionary Feature Construction (Kamath *et al.* 2014) has also been used to detect functional signals representing non-local, position-specific interactions at the nucleotide level (Veltri *et al.*, 2017).

Three recent papers have achieved improved model accuracies by applying Natural Language Processing (NLP) techniques to create vectorized embeddings of the amino acid sequences as the sole input features (Su *et al.*, 2019; Veltri *et al.*, 2018; Wu *et al.*, 2019). This has the benefit of reducing the time taken, as well as the expert biological knowledge needed, when creating model inputs. Furthermore, NLP approaches have the potential to represent richer positional information reflecting the specific locations of amino acids within a given sequence.

Veltri *et al.* (2018) utilized the Bag of Words (BoW) method to initially assign a unique numerical token to each amino acid in a sequence. This approach could recognize basic patterns, such as which amino acids are the same, and the frequency of specific amino acids in a sequence or throughout the dataset. However, BoW fails to capture component similarity. To partially alleviate this issue, an initial embedding layer was used in the neural network architecture, to convert the discrete vectors into a continuous and dense latent space represented by a three number vector, which could then reflect more complex relationships between inputs.

Su *et al.* (2019) built on this research by developing a multi-scale deep neural network (MS DNN), containing multiple convolutional layers, each using different filter lengths. This approach solely used an embedding layer to capture the semantic similarity between amino acids and found that long short-term memory (LSTM) layers did not improve the predictive ability of their model. Furthermore, Su *et al.* (2019) found that creating a fusion model by combining their MS DNN with traditional compositional methods further improved results.

Lastly, Wu *et al.* (2019) used the Word2Vec Skip-Gram algorithm to create embeddings. This method learns by being given a central word (or amino acid in this case) and then predicting the most likely words in a fixed window surrounding it, allowing it to reflect word similarity (Mikolov *et al.*, 2013). One drawback of all these approaches, however, is that they fail to convey the complex contextual information encoded by the position of each amino acid.

In contrast, this paper proposes a novel method of creating embedding vectors—by utilizing language representation models that have been pre-trained on large protein databases to produce *contextualized* embeddings. Language models (LMs) are built using the Transformer architecture (Vaswani *et al.*, 2017) and have primarily been used for language-based tasks, given that they have been pre-trained on large corpora such as the 2500 million words found in Wikipedia (Devlin *et al.*, 2019). They have been proven to outperform humans in some assessments, such as the Stanford Question Answering Dataset—a reading comprehension test (SQuAD 2.0, 2021), as well as the two NLP approaches previously mentioned. When these models are pre-trained on protein sequences, treating each amino acid as a word, and the sequence as a sentence, they have shown an ability to generalize towards understanding the ‘language of life’ itself, as Elnaggar *et al.* (2020) applied them to accurately predict both per-residue protein secondary structure and per-protein subcellular localization.

The LMs selected for this report are the auto-encoder models; Bidirectional Encoder Representations from Transformers (BERT; Devlin *et al.*, 2019) and Text-To-Text Transfer Transformer (T5; Raffel *et al.*, 2020), and the auto-regressive model XLNet (Yang *et al.*, 2020). These have already been pre-trained using the Summit supercomputer on either the UniRef100 or the UniRef50 datasets, consisting of 216 and 45 million protein sequences, respectively (Suzek *et al.*, 2015). The UniRef clusters are proteins sourced from the UniProt database, with the number referring to the similarity threshold set in the CD-HIT programme (Huang *et al.*, 2010). Therefore, UniRef100 contains all UniProt sequences, whereas UniRef50 only contains sequences that do not share more than 50% identity. Additionally, BERT and T5 were also pre-trained on the Big Fat Database dataset, comprising 2122 million sequences (Steinegger and Söding, 2018). All models were accessed via the ProtTrans Github page (https://github.com/agemagician/ProtTrans).

BERT aims to predict data from artificially corrupted inputs. It does this by adding [MASK] tokens during pre-training to replace a percentage of input words at random and it then aims to predict this masked word based on the surrounding context. In this manner, it is able to capture bi-directional context. However, all masked words are predicted in parallel and independently of one another, which means that some of the overall context of a sentence can be lost during training. Also, whilst the artificial [MASK] tokens are used during pre-training, they are absent when the model is subsequently fine-tuned on a specific task, which can result in a ‘pretrain-finetune discrepancy’ (Yang *et al.*, 2020).

XLNet is an auto-regressive model, which functions more similarly to a feed-forward neural network; these models aim to predict the next word from a set of words, given the context. However, unlike auto-encoder models, the prediction is constrained to be uni-directional. XLNet partially overcomes this drawback through permutation language modelling, whereby the model maximizes the expected log-likelihood of the sequence when all possible permutations of the sequence order are considered (Yang *et al.*, 2020). By learning context from randomly ordered sentences the resulting model is in essence bi-directional without requiring masking. Furthermore, XLNet utilizes a memory mechanism introduced by a previous auto-regressive model—Transformer-XL (Dai *et al.*, 2019)—which allows for the processing of longer contextual segments of data than BERT.

The T5 was introduced after Raffel *et al.* (2020) studied the current landscape of transfer learning techniques for NLP and found that, generally, encoder–decoder models outperformed those only utilizing one half of the Transformer architecture. The T5 model therefore uses both parts of the Transformer, in contrast to BERT which only uses the encoder, and XLNet which only uses the decoder. T5 was also trained on a new ‘Colossal Clean Crawled Corpus’, a comparatively *clean* and *natural* text dataset that was several magnitudes larger than many previous training datasets. There is evidence that this additional complexity, and the fact that positional encodings for each attention head can be shared across all layers, gives T5 a performance advantage over the other two LMs (Elnaggar *et al.*, 2021).

A convolutional neural network (CNN) has been chosen as the classifier based on its success in prior peptide prediction research (Veltri *et al.*, 2018; Wu *et al.*, 2019), as well as its successful application to other areas within bioinformatics, such as mapping protein sequences to folds (Hou *et al.*, 2018) or the prediction of RNA secondary structure (Zhang *et al.*, 2019). The convolutional layers apply filters that can interpret the spatial and temporal dependencies between amino acids that have been represented by the contextualized LM embeddings.

## 2 Methods

### 2.1 Datasets

Validation of the approach was performed on two datasets. One which was sourced externally and has been used in prior AMP prediction research—referred to as the ‘Veltri Dataset’. The second has been constructed independently using publicly available resources—the ‘LMPred Dataset’.

### 2.2 Veltri dataset

The Veltri dataset was sourced from the Antimicrobial Peptide Scanner web page (https://www.dveltri.com/ascan/). This contains 1778 AMP and 1778 non-AMP samples and is split into the exact training, validation and test sets that Veltri *et al.* (2018) used to build and evaluate their model.

### 2.3 LMPred dataset

The LMPred dataset has been built from a combination of external sources. The aim was to produce the largest and most up-to-date AMP dataset possible.

The positive samples (the AMPs) have been gathered from the freely available datasets shared by Veltri *et al.* (2018) and Bhadra *et al.* (2018) and combined with the peer-reviewed, natural AMPs taken from the DRAMP 2.0 database (Kang *et al.*, 2019). When duplicates were removed, these sources contributed 7053 AMPs. The samples are then filtered by removing AMPs <10 amino acids in length, as well as those sharing 90% sequence identity according to the CD-HIT online web server (Huang *et al.*, 2010). After filtering, the remaining 3758 AMPs were included in the LMPred dataset, being 24% more than the next largest collection used by Bhadra *et al.* (2018).

There is little incentive to experimentally prove a peptide is non-AMP, and thus there are no large repositories of peptides that have been shown to lack desirable activities. Therefore, without access to a formal non-AMP database, negative samples were collected similarly to prior research (Veltri *et al.*, 2018; Xiao *et al.*, 2013). Sequences that had been reviewed and verified were downloaded from the UniProt database (https://www.uniprot.org/). The data were then filtered according to the following criteria:

- Any duplicate entries were removed.
- Only samples whose subcellular location was cited as ‘cytoplasm’ were retained to ensure the origination was similar to the AMP samples.
- Any samples labelled as specifically having the activities: ‘antimicrobial’, ‘antibiotic’, antiviral’, ‘antifungal’, ‘effector’ or ‘excreted’ were omitted.
- Datapoints with fewer than 10 amino acids and more than 255 amino acids were removed. The remaining samples then mirrored the range of AMP sequence lengths in the positive sample dataset.
- Sequences which contained unnatural amino acids (Z, B, J, O, U or X) were removed.
- Finally, sequences were screened for similarity using the CD-HIT programme, using a 40% similarity threshold. The threshold can be stricter for non-AMPs given the larger number of available sequences, and this ensures a more diverse dataset overall.

Previous research found that if the negative sample sequence-length distribution matched that of the positive samples, this resulted in the highest classification accuracy models (Veltri *et al.*, 2018). Therefore, 3758 non-AMPs were selected from the remaining pool of 33 722 to compile a final dataset that matched this criterion. The dataset was then split using sklearn’s ‘train_test_split’ function (https://scikit-learn.org/); 40% of the data were set aside for training, 20% for validation and the remaining 40% for testing.

The Supplementary Information includes the sequence-length distributions of the AMP and non-AMP samples within the LMPred dataset. The splitting of train, test and validation sets was performed on a stratified basis to retain a similar distribution across all datasets. The data have been made freely available to download at https://github.com/williamdee1/LMPred_AMP_Dataset.

### 2.4 LM embeddings

Instructions were followed on the ProtTrans Github page for how to create word embeddings using each LM. An overview of this process is as follows:

- Download the specific tokenizer and pre-trained LM hosted on the ProtTrans Rostlab server.
- Convert the sequences of amino acids into a list and add spaces in between each amino acid.
- Any unnatural amino acids (‘U, Z, O, B, J’) are mapped to ‘X’.
- The sequence IDs are encoded in batches by the tokenizer, padding with zeros to a specified max length so all inputs are the same length.
- Torch tensors representing the input IDs and the mask used for the attention mechanism are created.
- The embeddings are generated in batches of 10 to ensure memory constraints are not breached and the output is saved as a numpy array.

Certain LMs produce special tokens, such as [CLS] or [SEP] tokens, that are included in the embedding array. The CLS token is created by some models to be used as an intelligent average one-dimensional vector summarizing the full two-dimensional embedding. It is often used as the input for NLP classification tasks. The SEP token separates any special tokens from the embeddings. In this project, the full embeddings were used as not all LMs produce CLS tokens and this ensures greater comparability across results. Additionally, using the full embeddings ensures valuable information is not lost and, instead, the neural network can decide how to screen the data through the use of the filters, kernels and max-pooling layers.

**Fig. 1.**
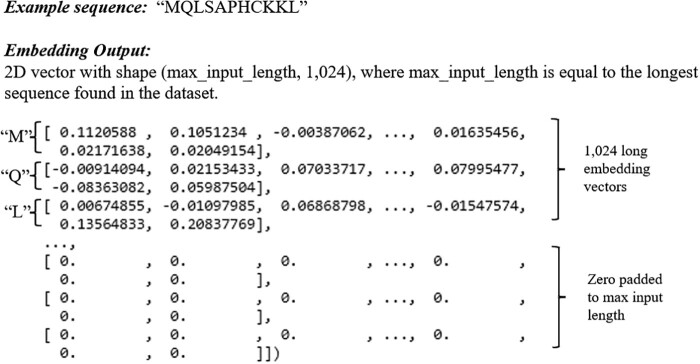
An example of how the sequence ‘MQLSAPHCKKL’ would be represented after applying a pre-trained LM to create an embedding vector

An example of a vectorized embedding produced by a pre-trained LM is shown in Figure 1.

### 2.5 Model architecture

A CNN was chosen as the classifier for this paper and was built using the Keras framework (https://keras.io/), which utilizes the Tensorflow (Abadi *et al.*, 2016) back-end.

**Fig. 2.**
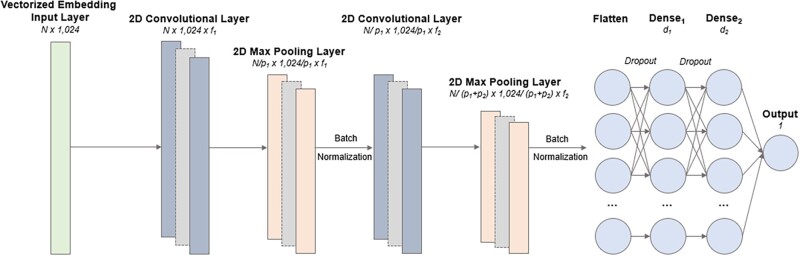
An illustration of the CNN architecture used in the best-performing LM embedding-based classifier—T5 Uniprot 50

Two different architectures were tested for each LM, altering the number of convolutional, max pooling, dense and batch normalization layers to investigate the impact on performance. These architecture changes were not found to significantly impact results, with the maximum difference being a 1.47% change in accuracy for the T5 model pre-trained on the Big Fat Database. Full results can be found within the Supplementary Information. The architecture selected for the best-performing LM—T5 UniRef50—can be found in Figure 2.

### 2.6 Hyperparameter tuning

For each set of LM embeddings, tuning was performed independently to ascertain the optimal hyperparameters for each resulting CNN model. The tuning was performed using the Keras Tuner (https://keras.io/keras_tuner), which provides a framework to apply different search algorithms. Both the Hyperband (Li *et al.*, 2016) and the Bayesian Optimization (Snoek *et al.*, 2012) tuning algorithms were used. Utilizing the two methods in combination has been shown to outperform using them separately as, ‘bandit-based approaches’ (like Hyperband) ‘lack guidance’, whereas Bayesian optimization across the entire search space can be ‘computationally infeasible’ (Falkner *et al.*, 2018). The tuning uses only the training and validation partitions of the data.

Tuning was performed for a maximum of 10 epochs for Hyperband, and 15 for Bayesian Optimization, with an early stopping call back after 6 epochs without a decrease in validation loss, and a reduction of the learning rate by a factor of 1e^−1^ after 4 epochs without a decrease. The optimal parameters were the ones, which produced the model with the lowest validation loss during training.

### 2.7 Model training and evaluation

Once hyperparameters were selected for each LM-embedding approach, each CNN model was trained for 30 epochs with the optimizer loss function set to ‘binary_crossentropy’ and metrics set to ‘accuracy’. Early stopping was set to 12 epochs, and the learning rate was reduced by a factor of 1e^−1^ after 4 epochs without an improvement in validation loss. The model checkpoint call back saved the model with the lowest validation loss during training.

In terms of evaluation, each model was tested for accuracy (Ac.) on the test set for both the Veltri and the LMPred datasets. Accuracy is considered the goal metric as identifying positive and negative samples correctly is equally important. Additionally, sensitivity (Sn.), specificity (Sp.), Matthew’s correlation coefficient (MCC) and the area under the ROC curve (AUC) were also calculated to provide a full overview of model performance. These metrics were calculated as follows using the number of true positive (TN), true negative (TN), false positive (FP) and false negative (FN) predictions:

(1) $$\text{Sn}.=\frac{\text{TP}}{\text{TP}+\text{FN}}$$

(2) $$\text{Sp}.=\frac{\text{TN}}{\text{TN}+\text{FP}}$$

(3) $$\text{Ac}.=\frac{\text{TP}+\text{TN}}{\text{TP}+\text{TN}+\text{FP}+\text{FN}}$$

(4) $$\text{MCC}=\frac{\left(\text{TP}\times \text{TN})-(\text{FP}\times \text{FN}\right)}{\sqrt{\left(\text{TP}+\text{FP})(\text{TP}+\text{FN})(\text{TN}+\text{FP})(\text{TN}+\text{FN}\right)}}.$$

The area under the ROC curve (AUC) was calculated using the sklearn metrics package.

The results were compared to that of the available webserver models, produced by previous state-of-the-art AMP prediction papers, when provided the same test sets. These include; the CAMP server’s artificial neural network, support vector machine, discriminant analysis and random forest models (Thomas *et al.*, 2010), AmPEP’s random forest (Bhadra *et al.*, 2018), iAMP-2L’s fuzzy K-nearest neighbour (Xiao *et al.*, 2013), iAMPpred’s support vector machine (Meher *et al.*, 2017) and Veltri’s CNN model (Veltri *et al.*, 2018).

### 2.8 Replicating Veltri’s prior approach

In order to ensure a robust comparison with Veltri *et al.*’s (2018) method for AMP prediction, a replica of their CNN was constructed. Firstly, it was tested on the Veltri dataset, and its efficacy was compared to that of the original paper, to verify it had been replicated correctly. This approach was then tested on the LMPred test set, having been trained using the additional training and validation samples provided by that dataset, which the Veltri webserver model has not had access to. Lastly, since some positive samples in the LMPred dataset were sourced from the dataset used by Veltri *et al.* (2018), the webserver model will have been trained on 457 AMPs it is then asked to predict. Metrics will therefore also be included showing the efficacy of the Veltri webserver model at predicting only unseen data.

### 2.9 Technical settings

Creating word embeddings, as well as hyperparameter tuning, training and evaluation of the CNN models was performed using Google Colab Pro (https://research.google.com/colaboratory/). A Tesla P100-PCIE-16 GB GPU was used, as well as up to 24 GB of RAM and 150 GB of disk resources.

Models using two convolutional layers (T5 UniRef50 and XLNet) took 107 s per epoch to train and 166 s to load and predict the 3007 test samples in the LMPred dataset. The size of those models was ∼1.16 GB. This is compared to 35 s per training epoch, 34 s for testing and 5.2 MB in size, for the models using one convolutional layer (BERT UniRef100, BERT BFD and T5 UniRef100).

## 3 Results

### 3.1 Veltri dataset

Table 1 shows the performance of the CNN models, using the different LM embeddings as inputs, when tested on the Veltri dataset. These results have been compared with the results published in Veltri *et al.*’s (2018) AMP peptide prediction paper, as well as Su *et al.*’s (2019) multi-scale DNN and fusion model results.

**Table 1.** Comparison with state-of-the-art methods—Veltri dataset

Approach	Method	Sn. (%)	Sp. (%)	Ac. (%)	MCC	AUC (%)
External models						
AntiBP2	SVM	87.91	90.80	89.37	0.7876	89.36
CAMP	ANN	82.98	85.09	84.04	0.6809	84.06
CAMP	DA	87.08	80.76	83.92	0.6797	89.97
CAMP	RF	**92.70**	82.44	87.57	0.7554	93.63
CAMP	SVM	88.90	79.92	84.41	0.6910	90.63
iAMP-2L	FKNN	83.99	85.86	84.90	0.6983	84.90
IAMPred	SVM	89.33	87.22	88.27	0.7656	94.44
gkmSVM	SVM	88.34	90.59	89.46	0.7895	94.98
Veltri	CNN	89.89	92.13	91.01	0.8204	96.48
Su	MS DNN	91.01	93.64	92.41	0.8486	97.23
Su	FUSION	89.89	94.96	92.55	0.8523	97.30
Models created by this paper						
Veltri (Replica)	CNN	88.48	92.70	90.60	0.8125	96.12
BERT (Uni 100)	CNN	90.31	93.82	92.06	0.8418	97.29
BERT (BFD)	CNN	91.99	92.13	92.06	0.8413	97.55
T5 (Uni 50)	CNN	92.28	94.38	**93.33**	**0.8668**	**97.89**
T5 (BFD)	CNN	88.62	**95.79**	92.21	0.8463	97.41
XLNet (Uni 100)	CNN	88.48	91.01	89.75	0.7952	95.78

*Notes:* The performance of the webserver models and the Veltri *et al.* model was sourced from the original Veltri *et al.* (2018) paper, whilst Su *et al.* (2019) was sourced directly. These results have been compared with the models built in this paper—being the replica of the Veltri model, as well as the models built using the different LM embeddings. The highest scoring model for each metric has been highlighted in **bold**.

The best-performing model—T5 pre-trained on the UniRef50 database—achieved an accuracy of 93.33%, being 0.84% higher than that Su *et al.*’s (2019) fusion model results of 92.55% on the same dataset. The T5 UniRef50 model also achieved state-of-the-art MCC and auROC metrics, only being bested on specificity by the T5 BFD model and on sensitivity by the CAMP Random Forest webserver model (Thomas *et al.*, 2010).

The auto-regressive model, XLNet, underperformed the two auto-encoder (BERT and T5) LM-based approaches, as well as the approach used by Veltri *et al.* (2018). This underperformance was noted across every metric. However, all models using NLP techniques to create embeddings, and CNNs as the classifier, displayed higher accuracies than those using compositional biological information and machine learning models.

### 3.2 LMPred dataset

Table 2 shows the performance of the CNN models when tested on the LMPred dataset, compared to the available webserver model predictions when provided the same test data. Similar results were found to Section 3.1, with the T5 auto-encoder LM, pre-trained on UniRef50, producing embeddings that resulted in the CNN with the highest accuracy (88.26%).

**Table 2.** Comparison with state-of-the-art methods—LMPred dataset

	Method	Sn. (%)	Sp.(%)	Ac.(%)	MCC	AUC (%)
External webserver models						
AmPEP	RF	56.62	43.48	50.05	0.1050	n/a^a^
CAMP	ANN	77.51	72.54	75.02	0.5011	n/a^a^
CAMP	DA	81.04	71.41	76.22	0.5269	80.16
CAMP	RF	84.70	72.74	78.72	0.5785	83.05
CAMP	SVM	83.03	71.88	77.45	0.5525	80.27
iAMP-2L	FKNN	76.18	74.60	75.39	0.5079	n/a^a^
IAMPred	SVM	89.55	55.92	72.73	0.4828	82.28
Veltri	CNN	**90.09**	66.95	78.52	0.5863	86.45
Veltri—Unseen Data^b^	CNN	86.42	66.95	74.94	0.5277	84.42
Models created by this paper						
Veltri (Replica)	CNN	75.98	85.11	80.55	0.6134	85.82
BERT (Uni 100)	CNN	84.43	84.91	84.67	0.6934	90.84
BERT (BFD)	CNN	86.83	88.16	87.50	0.7500	93.58
T5 (Uni 50)	CNN	88.89	87.63	**88.26**	**0.7653**	**94.66**
T5 (BFD)	CNN	86.16	**88.96**	87.56	0.7515	93.68
XLNet (Uni 100)	CNN	82.63	78.92	80.78	0.6160	88.87
Ten-fold cross-validation						
T5 (Uni 50)	CNN	88.66	85.54	87.12	0.7437	93.86

*Notes:* Results produced when testing models created within this paper, as well as external webserver models, on the LMPred test dataset. The highest scoring model for each metric has been highlighted in bold.

a The web server did not output prediction probabilities, so AUC could not be calculated.

b Refers to only the samples not already seen by the Veltri webserver model being submitted as the part of the LMPred test set.

The performance difference between the models created by this paper and the available web server models was more noticeable, likely due to the increased number of samples (111% more than the Veltri dataset) leading to a higher difficulty classification task. The T5 UniRef50 CNN produced 12.4% higher accuracy than the Veltri webserver model, which increased to a 17.8% gap when the Veltri webserver was only presented with unseen test data. Su *et al.* (2019) did not develop a web server model, meaning this dataset could not be tested with their fusion approach.

To differentiate between the impact of the increased number of training and validation samples, and the embedding approach taken, a replica of Veltri’s approach was built, utilizing the training and validation data of the LMPred dataset. This approach resulted in an uplift of 2.6% versus the Veltri webserver model, but still fell 9.6% short of the best LM-based CNN’s accuracy—implying that this is the true performance difference between approaches for this dataset.

Consistent with the results found for the Veltri dataset, all models leveraging NLP techniques and CNN’s scored higher accuracies than those utilizing specialist biological feature sets and traditional machine learning models. The T5 model trained on the BFD also displayed the highest specificity, whilst the Veltri and the IAMPred (Meher *et al.*, 2017) webserver models produced the highest sensitivity. The XLNet embeddings proved to be inferior to both BERT and T5 and did not perform significantly better than the replica Veltri model.

## 4 Discussion

This paper has proposed a novel method for producing model inputs for classifying AMPs. By utilizing the contextualized embeddings produced by pre-trained LMs, the resulting CNN achieved improved classification accuracy compared to the existing state-of-the-art methods across two datasets. This provides further evidence that NLP techniques can replicate some of the ‘language of life’, which previously required extensive time and biological knowledge in the feature engineering stages to represent.

The results support the research of Elnaggar *et al.* (2021) who found that true bi-directionality of contextual embedding was extremely important for protein structure prediction. This implies that the structure of amino acids is important bi-directionally and it is likely that the permutation modelling that XLNet uses, whilst it can work for sentences, is not so applicable in the case of peptide or protein sequences. The more distant dependency modelling that XLNet allows appears to be less beneficial in this use case, as the BERT-based embeddings do not suffer from lacking it.

Furthermore, this research also supported Elnaggar *et al.*’s (2021) results that found T5 to be the superior model for prediction—as the T5 model trained on UniRef50 generated the highest accuracy metric when tested on both datasets. This demonstrates the benefits of using the whole Transformer architecture to build the pre-trained LM, rather than just the encoder (BERT) or decoder (XLNet).

Also similar to Elnaggar *et al.*’s (2021) findings, there was no conclusive evidence that pre-training the LMs on larger datasets resulted in embeddings that improved predictive accuracy. On both the Veltri and the LMPred dataset, the T5 model trained on the BFD produced lower accuracy than the one trained on the UniRef50 dataset. The BERT BFD model did outperform the BERT UniRef100 model, but only on the larger, LMPred dataset. More diverse pre-training datasets may prove to be the optimal approach for this problem. This is implied by the results, as UniRef50 only includes the sequences from UniProt that do not share more than 50% sequence similarity. Additionally, the T5 model is also trained on a large, diverse ‘cleaned’ corpus, compared to the smaller and more uniform corpora used to initially produce BERT. The abundance of noise in these databases, i.e., in UniRef100 caused by duplicated sequences, may have proved detrimental to overall learning.

Future research could investigate the effectiveness of the pre-trained LMs not considered in scope by this paper. These include the ELECTRA and ALBERT models, as well as the Transformer-XL model when it is released on the ProtTrans Github page (https://github.com/agemagician/ProtTrans). This work would likely provide further support to the evidence found in this paper that auto-encoder models produce more context-rich embeddings, which can be more effectively used as inputs into CNN models predicting AMPs. It would be interesting to note whether these performance trends are similar across AMPs with differing activities (i.e. anticancer compared to antihypertensive peptides).

Further work could also split the AMPs into explicit cohorts, i.e. based on sequence length, or the frequency of specific amino acids, similar to Yan *et al.*’s (2020) paper predicting AMPs shorter than 30 amino acids in length. This approach may reveal that different LMs excel at predicting different cohorts, a hypothesis that may be supported by the evidence that longer-term dependency modelling is more important in the case of longer sentences for NLP tasks, or in this case—longer sequences. Lastly, Su *et al.* (2019) showed that a fusion model combining compositional information with deep learning embedding approaches can outperform using either method separately. It could therefore be productive to investigate the impacts of combining all the different approaches cited within this paper, to evaluate which combinations produces the optimal classification model.

## Supplementary Material

vbac021_Supplementary_Data

## References

[vbac021-B1] Abadi M. et al (2016) TensorFlow: a system for large-scale machine learning. In *Proceedings of the 12th USENIX Symposium on Operating Systems Design and Implementation (OSDI ’16),* p. 21.

[vbac021-B2] Bhadra P. et al (2018) AmPEP: sequence-based prediction of antimicrobial peptides using distribution patterns of amino acid properties and random forest. Sci. Rep., 8, 1697. 29374199 10.1038/s41598-018-19752-w PMC5785966

[vbac021-B3] Boman H.G. (2003) Antibacterial peptides: basic facts and emerging concepts. J. Intern. Med., 254, 197–215. 12930229 10.1046/j.1365-2796.2003.01228.x

[vbac021-B4] Chou K.C. (2001) Prediction of protein cellular attributes using pseudo-amino acid composition. Proteins, 43, 246–255. 11288174 10.1002/prot.1035

[vbac021-B5] Dai Z. et al (2019) Transformer-XL: attentive language models beyond a fixed-length context. *arXiv:1901.02860 [cs, stat]*. arXiv: 1901.02860. http://arxiv.org/abs/1901.02860 (27 February 2022, date last accessed).

[vbac021-B6] Devlin J. et al (2019) BERT: pre-training of deep bidirectional transformers for language understanding. *arXiv:1810.04805 [cs]*. arXiv: 1810.04805. http://arxiv.org/abs/1810.04805 (27 February 2022, date last accessed).

[vbac021-B7] Elnaggar A. et al (2020) ProtTrans: towards cracking the language of life’s code through self-supervised learning. *Bioinformatics*. http://biorxiv.org/lookup/doi/10.1101/2020.07.12.199554 (27 February 2022, date last accessed).

[vbac021-B8] Elnaggar A. et al (2021) ProtTrans: towards cracking the language of life’s code through self-supervised learning. *Technical report*. https://www.biorxiv.org/content/10.1101/2020.07.12.199554v3 (27 February 2022, date last accessed).

[vbac021-B9] Falkner S. et al (2018) BOHB: robust and efficient hyperparameter optimization at scale. *arXiv:1807.01774 [cs, stat]*. arXiv: 1807.01774. http://arxiv.org/abs/1807.01774 (27 February 2022, date last accessed).

[vbac021-B10] Fjell C.D. et al (2007) AMPer: a database and an automated discovery tool for antimicrobial peptides. Bioinformatics, 23, 1148–1155. 17341497 10.1093/bioinformatics/btm068

[vbac021-B11] Hou J. et al (2018) DeepSF: deep convolutional neural network for mapping protein sequences to folds. Bioinformatics, 34, 1295–1303. 29228193 10.1093/bioinformatics/btx780 PMC5905591

[vbac021-B12] Huang Y. et al (2010) CD-HIT Suite: a web server for clustering and comparing biological sequences. Bioinformatics, 26, 680–682. 20053844 10.1093/bioinformatics/btq003 PMC2828112

[vbac021-B13] Kamath U. et al (2014) Effective automated feature construction and selection for classification of biological sequences. PLoS One, 9, e99982. 25033270 10.1371/journal.pone.0099982 PMC4102475

[vbac021-B14] Kang X. et al (2019) DRAMP 2.0, an updated data repository of antimicrobial peptides. Sci. Data, 6, 148. 31409791 10.1038/s41597-019-0154-y PMC6692298

[vbac021-B15] Li F.-M. , Wang X.-Q. (2016) Identifying anticancer peptides by using improved hybrid compositions. Sci. Rep., 6, 33910. 27670968 10.1038/srep33910 PMC5037382

[vbac021-B16] Li L. et al (2016) Hyperband: a novel bandit-based approach to hyperparameter optimization. https://arxiv.org/abs/1603.06560v4 (27 February 2022, date last accessed).

[vbac021-B17] Manavalan B. et al (2017) MLACP: machine-learning-based prediction of anticancer peptides. Oncotarget, 8, 77121–77136. 29100375 10.18632/oncotarget.20365 PMC5652333

[vbac021-B18] Meher P.K. et al (2017) Predicting antimicrobial peptides with improved accuracy by incorporating the compositional, physico-chemical and structural features into Chou’s general PseAAC. Sci. Rep., 7, 42362. 28205576 10.1038/srep42362 PMC5304217

[vbac021-B19] Mikolov T. et al (2013) Efficient estimation of word representations in vector space. *arXiv:1301.3781 [cs]*. arXiv: 1301.3781. http://arxiv.org/abs/1301.3781 (27 February 2022, date last accessed).

[vbac021-B20] Raffel C. et al (2020) Exploring the limits of transfer learning with a unified text-to-text transformer. *arXiv:1910.10683 [cs, stat]*. arXiv: 1910.10683. http://arxiv.org/abs/1910.10683 (27 February 2022, date last accessed).

[vbac021-B21] Rayaprolu S.J. et al (2013) Peptides derived from high oleic acid soybean meals inhibit colon, liver and lung cancer cell growth. Food Res. Int., 50, 282–288.

[vbac021-B22] Rebucci M. , Michiels C. (2013) Molecular aspects of cancer cell resistance to chemotherapy. Biochem. Pharmacol., 85, 1219–1226. 23435357 10.1016/j.bcp.2013.02.017

[vbac021-B23] Snoek J. et al (2012) Practical Bayesian optimization of machine learning algorithms. In: *Advances in Neural Information Processing Systems*, Vol. 25. Curran Associates, Inc. https://proceedings.neurips.cc/paper/2012/hash/05311655a15b75fab86956663e1819cd-Abstract.html (27 February 2022, date last accessed).

[vbac021-B24] SQuAD 2.0. (2021) The Stanford Question Answering Dataset. https://rajpurkar.github.io/SQuAD-explorer/ (27 February 2022, date last accessed).

[vbac021-B25] Steinegger M. , Söding J. (2018) Clustering huge protein sequence sets in linear time. *Nat. Commun.* https://www.nature.com/articles/s41467-018-04964-5 (27 February 2022, date last accessed). 10.1038/s41467-018-04964-5 PMC6026198 29959318

[vbac021-B26] Su X. et al (2019) Antimicrobial peptide identification using multi-scale convolutional network. BMC Bioinformatics, 20, 730. 31870282 10.1186/s12859-019-3327-y PMC6929291

[vbac021-B27] Suzek B.E. , et al; UniProt Consortium. (2015) UniRef clusters: a comprehensive and scalable alternative for improving sequence similarity searches. Bioinformatics (Oxford, England), 31, 926–932. 25398609 10.1093/bioinformatics/btu739 PMC4375400

[vbac021-B28] Thomas S. et al (2010) CAMP: a useful resource for research on antimicrobial peptides. Nucleic Acids Res., 38, D774–D780. 19923233 10.1093/nar/gkp1021 PMC2808926

[vbac021-B29] Thundimadathil J. (2012) Cancer treatment using peptides: current therapies and future prospects. J. Amino Acids, 2012, e967347. 10.1155/2012/967347 PMC3539351 23316341

[vbac021-B30] Tyagi A. et al (2013) In silico models for designing and discovering novel anticancer peptides. Sci. Rep., 3, 2984. 24136089 10.1038/srep02984 PMC6505669

[vbac021-B31] Vaswani A. et al (2017) Attention is all you need. *arXiv:1706.03762 [cs]*. arXiv: 1706.03762. http://arxiv.org/abs/1706.03762 (27 February 2022, date last accessed).

[vbac021-B32] Veltri D. et al (2017) Improving recognition of antimicrobial peptides and target selectivity through machine learning and genetic programming. IEEE/ACM Trans. Comput. Biol. Bioinform., 14, 300–313. 28368808 10.1109/TCBB.2015.2462364

[vbac021-B33] Veltri D. et al (2018) Deep learning improves antimicrobial peptide recognition. Bioinformatics, 34, 2740–2747. 29590297 10.1093/bioinformatics/bty179 PMC6084614

[vbac021-B34] Wang A. et al (2014) Identification of stem-like cells in non-small cell lung cancer cells with specific peptides. Cancer Lett., 351, 100–107. 24846302 10.1016/j.canlet.2014.05.004

[vbac021-B35] World Health Organization. (2014) Antimicrobial Resistance: Global Report on Surveillance. World Health Organization, Geneva, Switzerland.

[vbac021-B36] Wu C. et al (2019) PTPD: predicting therapeutic peptides by deep learning and word2vec. BMC Bioinformatics, 20, 456. 31492094 10.1186/s12859-019-3006-z PMC6728961

[vbac021-B37] Xiao X. et al (2013) iAMP-2L: a two-level multi-label classifier for identifying antimicrobial peptides and their functional types. Anal. Biochem., 436, 168–177. 23395824 10.1016/j.ab.2013.01.019

[vbac021-B38] Yan J. et al (2020) Deep-AmPEP30: improve short antimicrobial peptides prediction with deep learning. Mol. Ther. Nucleic Acids, 20, 882–894. 32464552 10.1016/j.omtn.2020.05.006 PMC7256447

[vbac021-B39] Yang Z. et al (2020) XLNet: generalized autoregressive pretraining for language understanding. *arXiv:1906.08237 [cs]*. arXiv: 1906.08237. http://arxiv.org/abs/1906.08237 (27 February 2022, date last accessed).

[vbac021-B40] Zhang H. et al (2019) A new method of RNA secondary structure prediction based on convolutional neural network and dynamic programming. Front. Genet., 10, 467. 31191603 10.3389/fgene.2019.00467 PMC6540740

