# Supplementary material for: LMPred: predicting antimicrobial peptides using pre-trained language models and deep learning
Source: Bioinform Adv. 2022 Mar 31;2(1):vbac021. doi: 10.1093/bioadv/vbac021 (PMC9710646; doi:10.1093/bioadv/vbac021)
Supplement: vbac021_Supplementary_Data [file vbac021_supplementary_data.pdf]

LMPred: Predicting Antimicrobial Peptides Using Pre-Trained Language Models and Deep Learning

Supplementary Information

William Dee

1. Sequence Length Distributions – LMPred Dataset:

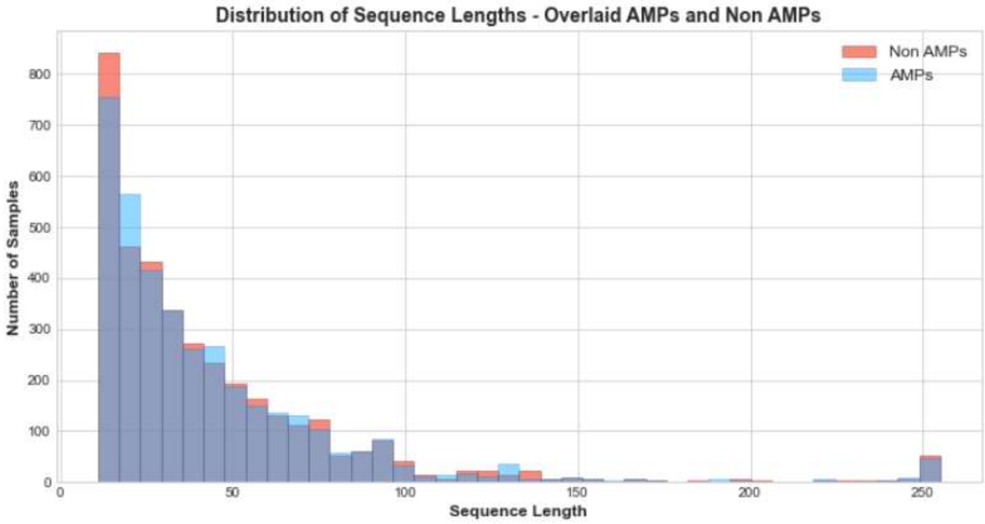

Figure S1: Sequence length distributions of the 3,758 AMP (blue) and 3,758 non-AMP (red) samples in the full LMPred dataset.

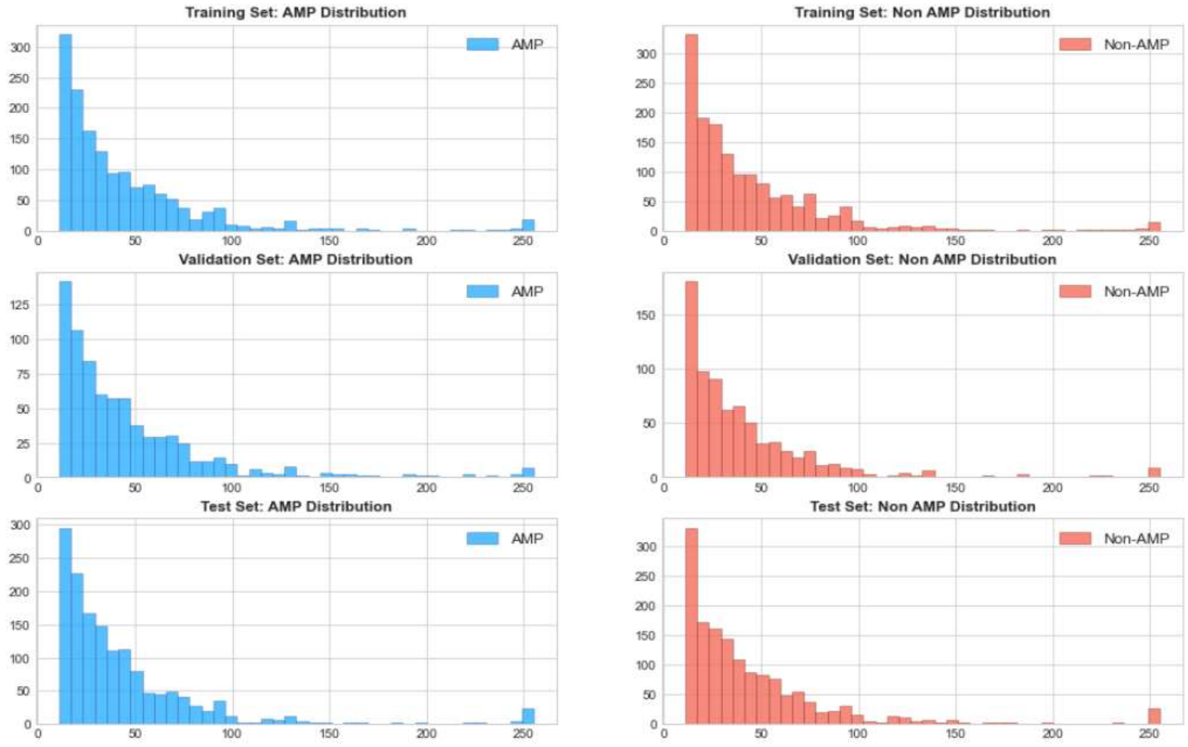

Figure S2: Sequence length distributions of the AMPs and non-AMPs across the training (40%), validation (40%) and test (20%) datasets.

2. Mean Embedding Values

Below shows some evidence of the different information conveyed by the embeddings produced by the different pre-trained language models. This is most obvious when looking at the embedding of "L" in position 11 of the non-AMP sample, compared to it's embedding in position 10 of the AMP sample. This difference is most stark for the T5 models where "L" is given the average value of -0.002 in the non-AMP and -0.010 in the AMP position. Overall, the T5 models appear to display the most absolute variance between embeddings.

Non-AMP Sequence

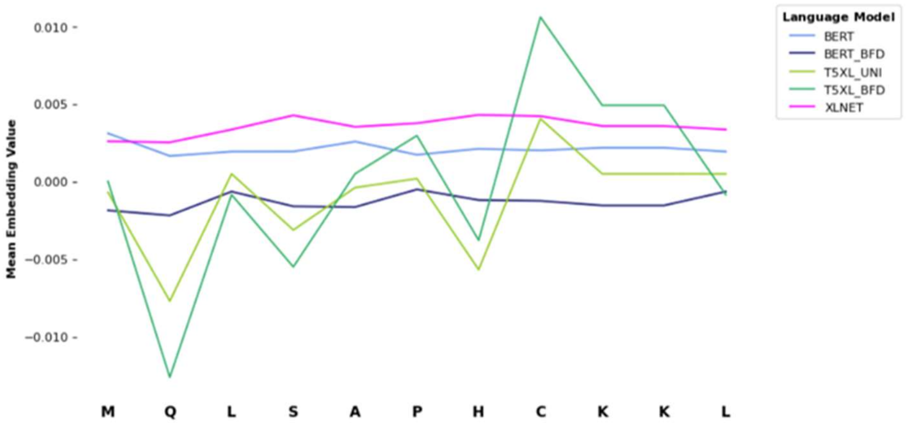

AMP Sequence

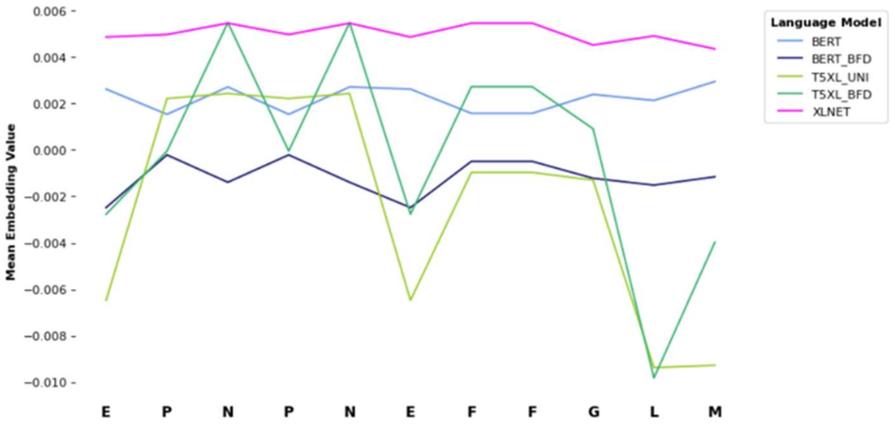

Figure S3: Line plots showing the comparative average value per amino acid assigned by the different pre-trained language models to an AMP and non-AMP 11 amino acids in length.

3. Comparisons of Model Architectures on Veltri Dataset

Table 1 shows the accuracy metrics achieved for all language models tested when using either a basic configuration with one convolutional, one max pooling, one batch normalization and then a dense sigmoidal output layer, or a more complex architecture employing two convolutional, two max pooling, two batch normalization, two dense and two dropout layers.

These architecture setups were tested on the Veltri Dataset and the results of the best performing architectures – being 1 layer for BERT Uni 100, BERT BFD and T5 BFD, and 2 layer for T5 Uni 50 and XLNet – were reported for both datasets in the Results section.

Language Model	Pre-Train Dataset	Architecture - Layers	Accuracy (%)
BERT	Uniref 100	1	92.06
BERT	Uniref 100	2	91.71
BERT	Big Fat Database	1	92.06
BERT	Big Fat Database	2	91.50
T5	Uniref 50	1	92.70
T5	Uniref 50	2	93.33
T5	Big Fat Database	1	92.21
T5	Big Fat Database	2	90.87
XLNet	Uniref 100	1	88.76
XLNet	Uniref 100	2	89.75

Table 1: Performance comparison for each language model method when using different architectural setups.

4. Data Availability

The LMPred dataset, along with notebooks detailing how the research can be replicated, can be found at the following Github page:

https://github.com/williamdee1/LMPred_AMP_Dataset
